# Supplementary material for: Scaffolding and completing genome assemblies in real-time with nanopore sequencing
Source: Nat Commun. 2017 Feb 20;8:14515. doi: 10.1038/ncomms14515 (PMC5321748; doi:10.1038/ncomms14515)
Supplement: Supplementary Information — Supplementary Figures 1-5 and Supplementary Table 1 [file ncomms14515-s1.pdf]

Supplementary Figures

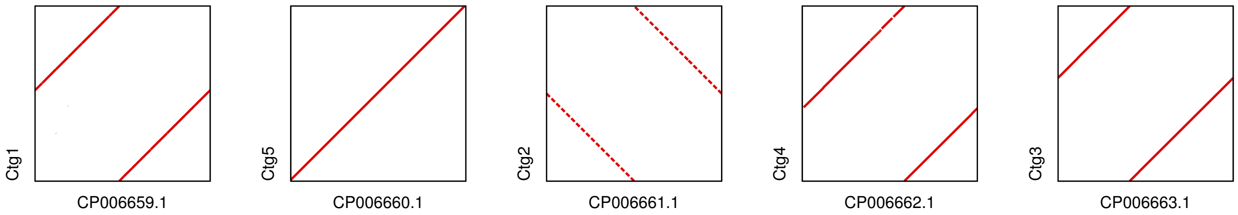

Supplementary Figure 1. Alignment of the npScarf's assembly for *K. pneumoniae* ATCC BAA-2146 to its draft reference genomes (GeneBank Accession GCA_000364385.2). The five contigs were in complete agreement with the chromosome and four plasmids.

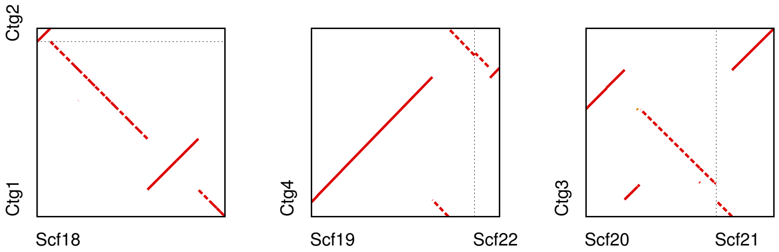

Supplementary Figure 2. Alignment of the npScarf's assembly for *K. pneumoniae* ATCC 13883 to its draft reference genomes (GeneBank Accession GCA_000742135.1). For display convenience, IDs of reference sequences are abbreviated by the last two digits before the dot in the original accession code: Contigs 1 and 2 were aligned to the reference Scaffold 18 (KN046818.1). Contig 4 was aligned to two Scaffolds 19 (KN046819.1) and 22 (KN046822.1) and Contig 3 to Scaffolds 20 (KN046820.1) and 21 (KN046821.1).

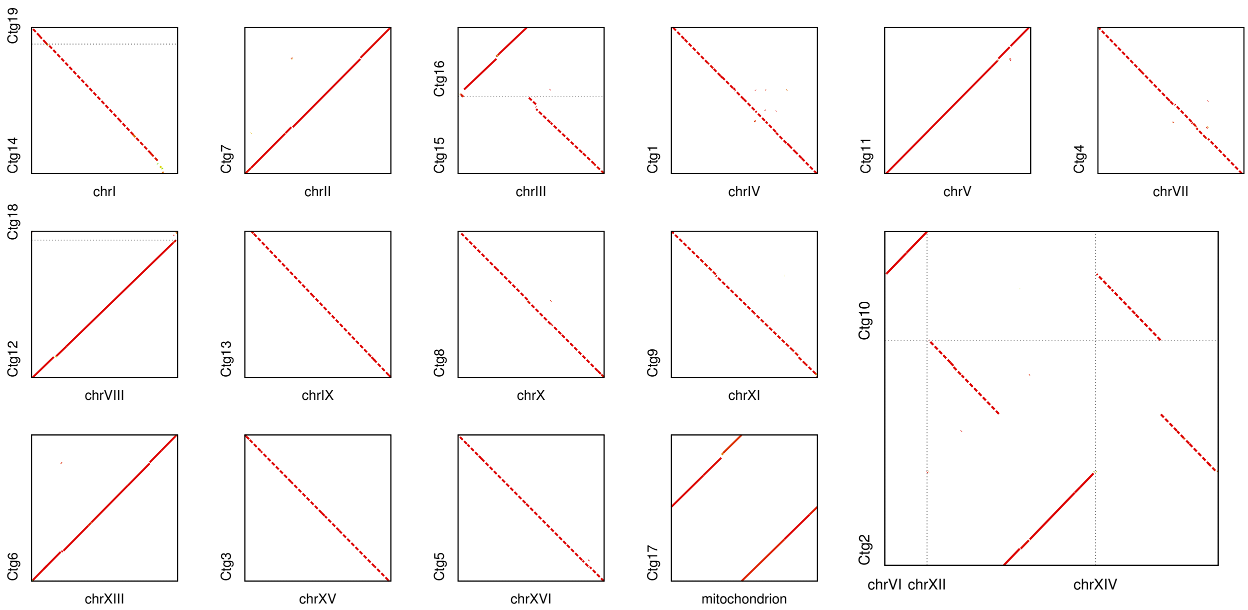

Supplementary Figure 3. Alignment of the npScarf's assembly for *S. cerevisae* W303 to the reference genome of the S288C strain. Ten chromosomes (II, IV, V, VII, IX, X, XI, XIII, XV, XVI and the mitochondrion) were constructed into individual contigs, three chromosomes (I, III and VIII) were into two contigs each, and three chromosomes (VI, XII and XIV) were fused into two contigs because of mis-assembly.

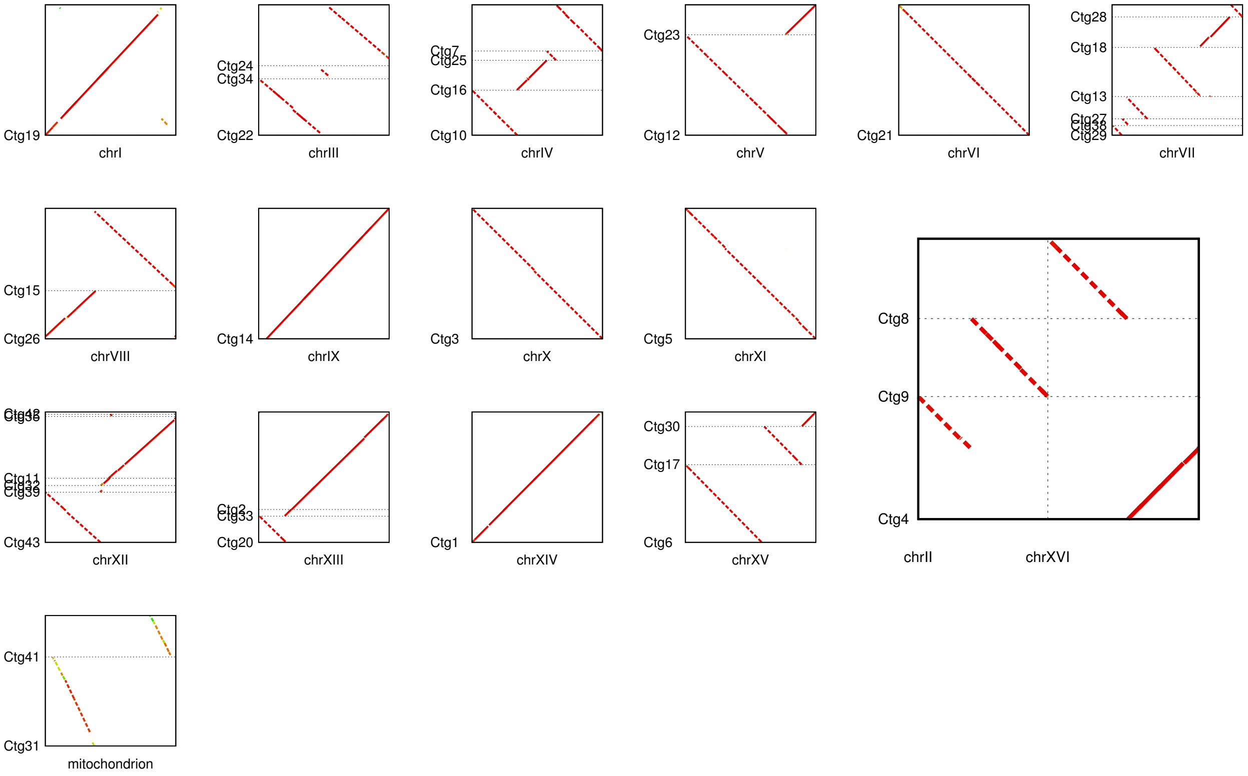

Supplementary Figure 4. Alignment of the Canu's assembly for *S. cerevisae* W303 to the reference genome of the S288C strain. Chromosomes II and XVI were fused onto three contigs.

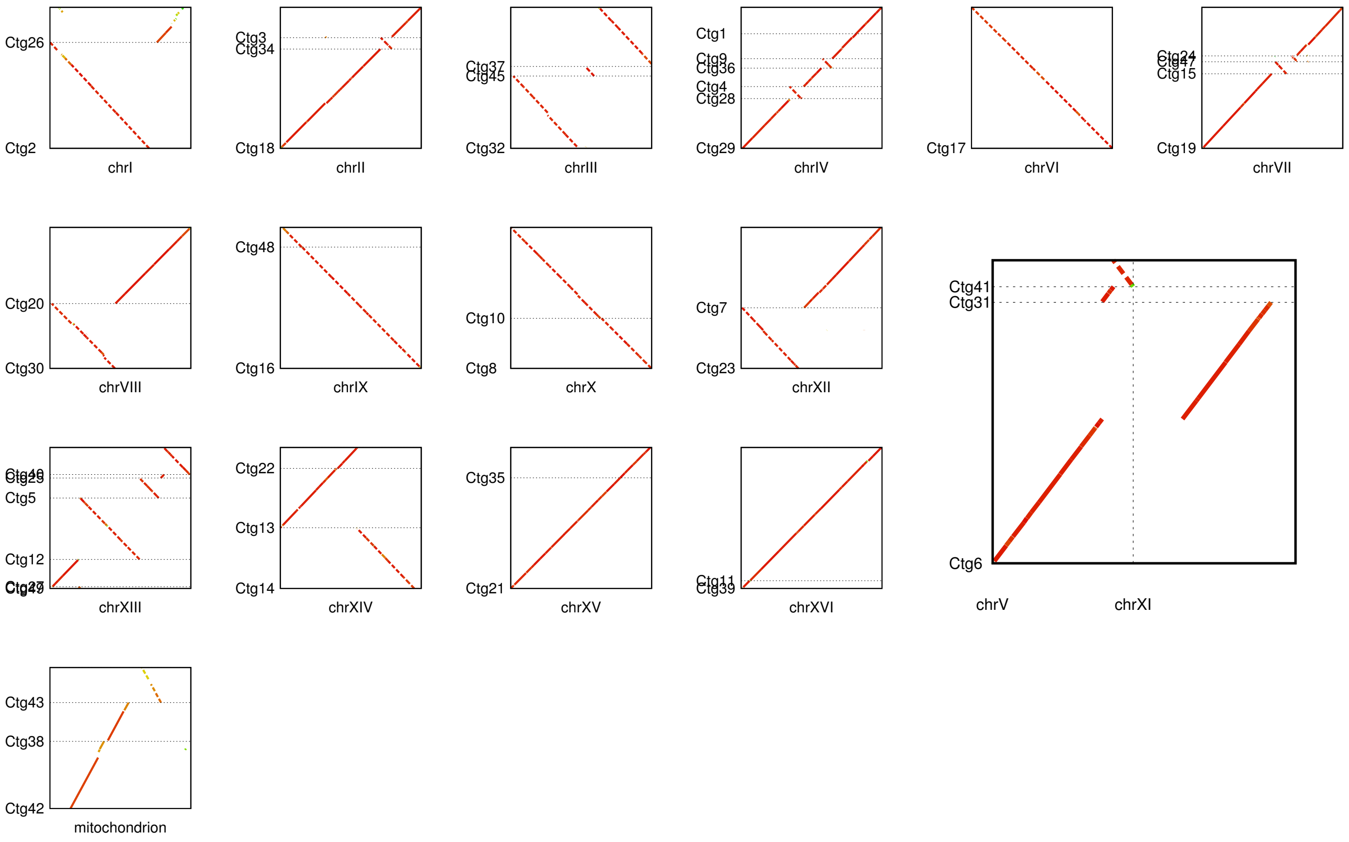

Supplementary Figure 5. Alignment of the miniasm's assembly for *S. cerevisae* W303 to the reference genome of the S288C strain. Chromosomes V and XI were fused onto three contigs.

Supplementary Table

Supplementary Table 1. Memory usage (Gb) of the different tools

	Kp2146	Kp13883	E.coli K12	ST H58	Sc W303
SPAdes	35.67	34.32	34.24	10.45	85.99
+ SSPACE	3.41	4.01	5.09	2.39	36.84
+ LINK	28.47	16.33	44.83	19.89	233.49
+ npScarf (rt)	2.11	1.11	2.32	1.27	4.27
NaS + CA	8.02	8.10	9.21	8.23	83.60
Nanocorr + CA	3.76	3.99	6.91	1.57	159.91
Canu + Pilon	-	6.78	6.30	-	56.20
Miniasm + Pilon	2.99	6.08	6.04	1.97	89.51

Supplementary Table 1 shows the memory usage of the tools on the datasets described in the paper. The scaffolders (SSPACE, LINK and npScarf) were run on the short read assemblies outputed by SPAdes. We hence only present the memory footprint from running these tools only. The memory requirement for each pipeline should be the *maximum* between memory usage of the tool and SPAdes. For NaS and Nanocorr, the error correction steps were distributed across hundreds of jobs, each consumed a small memory footprint. We report here only the memory usage from running Celera Assembler. Note that we ran Celera Assembler on differing configurations, and we reported the here the memory usage of the configuration resulting in the most complete assembly. Memory reported for Canu and Miniasm was the *maximum* among all tasks in the pipeline (including Pilon and BWA-MEM) because each task can be run one at a time; However, the reported memory usage for npScarf was the *sum* of memory for running npScarf and BWA-MEM.
